# Impact of donor transaminases on liver transplant utilisation and unnecessary organ discard: national registry cohort study

**DOI:** 10.3389/frtra.2024.1458996

**Published:** 2024-09-04

**Authors:** Joseph J. Dobbins, Samuel J. Tingle, Jennifer Mehew, Emily R. Thompson, Georgios Kourounis, Stuart McPherson, Steve A. White, Colin H. Wilson

**Affiliations:** ^1^ Institute of Transplantation, Freeman Hospital, Newcastle upon Tyne, United Kingdom; ^2^ National Institute for Health Research Blood and Transplant Research Unit (NIHR BTRU) in Organ Donation and Transplantation, Institute of Transplantation, Freeman Hospital, Newcastle upon Tyne, United Kingdom; ^3^ Translational and Clinical Research Institute, Newcastle University, Newcastle upon Tyne, United Kingdom; ^4^ Statistics and Clinical Research Department, NHS Blood and Transplant (NHSBT), Bristol, United Kingdom; ^5^ Department of Hepatology, Freeman Hospital, Newcastle upon Tyne, United Kingdom

**Keywords:** liver transplant, utilisation, organ decline, transaminases, ALT

## Abstract

**Background:**

Donor liver transaminases (ALT and AST) have been used to decline livers for transplant, despite evidence that they do not influence transplant outcomes. This study assesses the effect that raised donor transaminases have on the unnecessary decline of livers.

**Methods:**

This retrospective cohort study used the National Health Service registry on adult liver transplantation (2016–2019). Logistic regression models were built to assess the impact of donor transaminases on the utilisation of organs donated following brain stem death (DBD) and circulatory death (DCD). A further model was used to simulate the impact on liver decline if raised donor ALT was not used to make utilisation decisions.

**Results:**

5,424 adult livers were offered for transplant, of which 3,605 were utilised (2,841 DBD, 764 DCD). In multivariable analysis, adjusted for key factors, increasing peak donor ALT independently increased the odds of liver decline (DBD aOR = 1.396, 1.305–1.494, *p* < 0.001, DCD aOR = 1.162, 1.084–1.246, *p* < 0.001). AST was also a significant predictor of liver decline. 18.5% of livers from DBD donors with ALT > 40 U/L (*n* = 1,683) were declined for transplantation. In this group, our model predicted a 48% (38%–58%) decrease in decline if raised donor ALT was excluded from these decisions. This represents an additional 37 (30–45) liver transplants every year in the UK.

**Conclusions:**

Raised donor ALT increased the likelihood of liver decline. As it does not influence transplant outcome, avoiding donor ALT-based organ decline is an immediate and effective way to expand the donor pool.

## Introduction

Liver transplantation is the definitive treatment for end stage liver failure. Expanding the number of livers available for transplant remains a global priority. Currently, up to 50% of livers offered for transplantation in the UK are declined (1).

The decision to decline a liver is based on a range of donor factors, one of which is often liver serum transaminases. Liver transaminases (ALT and AST) are readily measured in the hospital setting and represent a marker of acute hepatocyte damage. Different clinicians have varying thresholds for donor ALT. Some will decline a liver based on small elevations, some see it as an unimportant factor and will accept livers from donors with very raised ALT. Therefore, grafts have been transplanted with a wide range of ALT values. Previous work on UK and US registry data has leveraged this retrospective data and shown no relationship between donor transaminases and transplant outcome (2, 3). This work demonstrated with a high degree of certainty that moderate elevations in donor ALT (ALT < 1,000 U/L) are not important predictors of poor transplant outcomes. In addition, several studies have reported successful liver transplants from donors with extreme elevations in transaminase levels (4, 5).

As transaminases do not predict transplant outcome, our recent recommendation was to avoid their use in utilisation decision making (2). Here we assess the effect that the perceived risk of raised serum transaminases had on liver decline, over this same period and estimate the potential number of additional livers which could be utilised if physicians avoided declining livers based on raised transaminases.

## Materials and methods

National UK registry data on livers offered for transplantation collected and validated by the National Health Service Blood and Transplant (NHSBT) were reviewed. We included actual organ donors (organ donors where at least one organ was transplanted) where the liver was offered for transplantation between 1 January 2016 and 31 December 2019. These dates represent the same period for which our previous work showing no effect of transaminases on transplant outcome was conducted (2). Donors aged <16 years were excluded. No serial transaminase data were recorded prior to this time and the subsequent years were disrupted by the COVID 19 pandemic.

### Statistical analysis

Missing data are summarised in Supplementary Table S1. To deal with missing data we employed multiple imputation (fully conditional specification) to produce 5 imputed data sets. All transaminase data were log transformed prior to multiple imputation to adjust for significant right skew.

Our primary aim was to assess the effect of ALT on liver decline rate. We also investigated AST, however this is less specific to liver injury and less widely performed in the UK. There is far more missing data for AST compared with ALT. To assess the independent effect of transaminases we needed to adjust for all other significant predictors of liver decline. Factors that may affect transplant outcome were identified from the data available and from validated acceptance criteria. Multiple logistic regression with backward stepwise selection was then performed to identify key variables. Variables with a significant effect on liver decline in 4–5 of the imputed data sets were included in the final adjusted multivariable model using pooled data from all 5 imputations.

Separate models were built for livers donated after brain death (DBD) and circulatory death (DCD) donors, to allow for assessment of the effect of normothermic regional perfusion (NRP) in the DCD group. A third model combined all the variables identified in the DBD and DCD models along with a donor type variable, to assess liver decline across all donor types. Results are displayed as adjusted odds ratios (aOR) and 95% confidence intervals. Additional models were built using restricted cubic splines (3 knots, 10/50/90th percentiles) to visualise the relationship between ALT and utilisation without assuming linear relationships.

We repeated these analyses for AST. As there is significant correlation between ALT and AST, each was examined in a separate model to avoid issues with collinearity. A further model was also built to assess the dichotomised variables of “normal ALT” and “raised ALT”.

### Modelling the impact of ALT on organ decline decisions

Primary reason for organ decline is often poorly recorded, and raised ALT may contribute to a decision to decline a liver without being the primary reason for decline; we therefore developed a new methodology to model how utilisation would be affected if raised donor ALT values were ignored.

Different clinicians may have different thresholds for when ALT effects their decision to accept a liver. However, we can be sure that no livers are being rejected based on an ALT < 40 as this is within the normal range reported by most biochemistry laboratories. We therefore dichotomised our data into “normal ALT” (donor ALT ≤ 40) and “raised ALT” (donor ALT > 40) groups.

The main logistic regression models for DCD and DBD organ decline (described above) were then taken, donor ALT was removed from the model, and the logistic regression model effect estimates were re-calculated using the “normal ALT” group. This was done separately for DBD and DCD. This predictive model reflects the probability of a liver being declined (taking into account all key factors, identified in previous modelling) when the ALT value is normal (and therefore not being used in the decision-making process). We then applied these models to the “raised ALT” DBD and DCD groups. This allowed us to estimate the predicted probability of decline for each individual liver with raised ALT; this prediction relates to the probability of decline were that donor to instead have normal ALT, and accounts for all donor factors which are important in utilisation. Differences between the predicted utilisation rate (were the ALT to be normal) and the actual utilisation rate (number of livers transplanted) in the “raised ALT” groups were calculated. This difference between actual and predicted decline rate allowed us to estimate the number of livers with raised ALT that would have been accepted for transplant if ALT were ignored. As before, separate models were run for DBD and DCD groups as predictors of decline differ in these groups. This technique assumes that predictions from the model built within the normal ALT cohort (which does not include ALT as a factor) are generalisable to the raised ALT cohort. i.e., that differences in predicted risk of decline and actual decline rate in the raised ALT cohort are solely the result of the ALT being raised. We feel that this is a valid assumption as the two cohorts are from the exact same time span and were being assessed by the same group of surgeons. 95% confidence interval was generated using the bootstrapping method (percentile method with 1,000 bootstrapped samples). This gives the uncertainty in the difference between predicted and actual utilisation rates, with the assumption that the model for predicting utilisation probability is appropriate. We feel this assumption is valid as the model predicting utilisation was trained on donors with peak ALT < 40 over the exact same time-period, and these offers were assessed (and utilisation decisions were made) by the exact same clinicians.

Whilst previous research has demonstrated no association between donor ALT and outcome, it remains uncertain whether extreme elevations in donor ALT (>1,000 U/L) predict outcome, due to low numbers of transplanted livers in this group. At ALT > 1,000 confidence intervals sharply increase when looking at impact on graft survival (2). We therefore carried out sensitivity analyses running our models using only the livers with ALT values less than 1,000 U/L to ensure this did not have a significant effect on the results.

For all tests performed, a *P* value of <0.05 was deemed significant. Analyses were performed in SPSS version 26 (IBM Corp, Armonk, NY) and R (R Foundation for Statistical Computing, Vienna, Austria).

## Results

5,424 adult liver donors (3,350 DBD, 2,074 DCD) were offered for transplant and included in our analysis. 3,605 of these livers were accepted and utilised (2,841 DBD, 84.8%; 764 DCD, 36.8%). Cohort demographics are summarised in Table 1.

**Table 1:** Cohort demographics.

Variable	DBD (*N* = 3,350)	DCD (*N* = 2,074)	Overall (*N* = 5,424)
Normothermic regional perfusion			
No NRP	3,350 (100%)	1,942 (93.6%)	5,292 (97.6%)
NRP	0 (0%)	132 (6.4%)	132 (2.4%)
Donor cause of death			
CVA	2,177 (65.0%)	854 (41.2%)	3,031 (55.9%)
Trauma	103 (3.1%)	71 (3.4%)	174 (3.2%)
Hypoxia	767 (22.9%)	870 (41.9%)	1,637 (30.2%)
Other	303 (9.0%)	279 (13.5%)	582 (10.7%)
Donor age (years)			
Median [Min, Max]	53.0 [16.0, 84.0]	56.0 [16.0, 83.0]	54.0 [16.0, 84.0]
Donor sex			
Male	1,718 (51.3%)	1,290 (62.2%)	3,008 (55.5%)
Female	1,632 (48.7%)	784 (37.8%)	2,416 (44.5%)
Donor BMI (kg/m^2^)			
Median [Min, Max]	26.2 [13.7, 49.5]	26.6 [13.6, 48.9]	26.3 [13.6, 49.5]
Year of donation			
2016	733 (21.9%)	510 (24.6%)	1243 (22.9%)
2017	817 (24.4%)	489 (23.6%)	1,306 (24.1%)
2018	921 (27.5%)	508 (24.5%)	1,429 (26.3%)
2019	879 (26.2%)	567 (27.3%)	1,446 (26.7%)
Donor blood group			
O	1,629 (48.6%)	941 (45.4%)	2,570 (47.4%)
A	1,303 (38.9%)	890 (42.9%)	2,193 (40.4%)
B	312 (9.3%)	171 (8.2%)	483 (8.9%)
AB	106 (3.2%)	72 (3.5%)	178 (3.3%)
History of cardiac disease			
No Hx cardiac disease	2,949 (88.0%)	1,736 (83.7%)	4,685 (86.4%)
Hx cardiac disease	401 (12.0%)	338 (16.3%)	739 (13.6%)
History of hypertension			
No Hx hypertension	2,297 (68.6%)	1,408 (67.9%)	3,705 (68.3%)
Hx hypertension	1,053 (31.4%)	666 (32.1%)	1,719 (31.7%)
History of diabetes			
No Hx diabetes	3,085 (92.1%)	1,895 (91.4%)	4,980 (91.8%)
Hx diabetes	265 (7.9%)	179 (8.6%)	444 (8.2%)
Family history of diabetes			
No FHx diabetes	2,267 (67.7%)	1,431 (69.0%)	3,698 (68.2%)
FHx diabetes	1,083 (32.3%)	643 (31.0%)	1,726 (31.8%)
History of liver disease			
No Hx liver disease	3,277 (97.8%)	2,019 (97.3%)	5,296 (97.6%)
Hx liver disease	73 (2.2%)	55 (2.7%)	128 (2.4%)

DBD, donation after brain death; DCD, donation after circulatory death; NRP, normothermic regional perfusion; CVA, cerebrovascular accident.

### The effect of donor ALT on liver decline

Table 2 displays the independent risk factors associated with liver decline. Donor max ALT has a significant independent effect on liver decline in adjusted multiple logistic regression models in both DBD and DCD donors (Table 2). A doubling in donor ALT is associated with a 39.6% increase in odds of decline of DBD livers and a 16.2% increase in odds of decline of DCD livers (DBD aOR = 1.396 (95% CI: 1.305–1.494) *p* < 0.001, DCD aOR = 1.162 (95% CI: 1.084–1.246) *p* < 0.001). A model including the entire cohort supported these findings [Supplementary Table S2, aOR = 1.279 (95% CI: 1.218–1.342) *p* < 0.001].

**Table 2:** Multiple logistic regression showing the effect of donor ALT on liver decline in DBD and DCD donors.

Variable	DBD		DCD	
	Adjusted OR (95% CI)	*P* value	Adjusted OR (95% CI)	*P* value
Donor max ALT (Log_2_ALT)^a^	1.396 (1.305–1.494)	**<0**.**001**	1.162 (1.084–1.246)	**<0**.**001**
Cause of death				
CVA	1	–	1	–
Trauma	0.850 (0.457–1.581)	0.608	0.886 (0.521–1.505)	0.654
Hypoxia	0.448 (0.333–0.604)	**<0**.**001**	1.134 (0.892–1.442)	0.303
Other	0.779 (0.537–1.129)	0.188	1.781 (1.289–2.459)	**<0**.**001**
Donor age (years)	1.020 (1.012–1.028)	**<0**.**001**	1.023 (1.016–2.459)	**<0**.**001**
Donor sex				
Male	1	–	–	–
Female	0.770 (0.626–0.947)	**0**.**013**	–	–
Donor BMI (kg/m^2^)	1.080 (1.060–1.101)	**<0**.**001**	1.099 (1.078–1.121)	**<0**.**001**
Year of donation (years)	1.164 (1.061–1.276)	**0**.**001**	1.158 (1.063–1.261)	**<0**.**001**
Donor blood group				
O	1	–	1	–
A	1.097 (0.881–1.367)	0.407	1.225 (0.999–1.502)	0.051
B	1.289 (0.910–1.825)	0.152	1.169 (0.811–1.685)	0.403
AB	4.310 (2.723–6.821)	**<0**.**001**	2.572 (1.386–4.773)	**0**.**003**
History of diabetes	1.745 (1.259 -2.419)	**<0**.**001**	–	–
Family history of diabetes	1.367 (1.101–1.697)	**0**.**005**	–	–
History of liver disease	6.741 (4.061–11.188)	**<0**.**001**	2.641 (1.304–5.351)	**0**.**007**
History of cardiac disease	–	–	1.447 (1.078–1.943)	**0**.**014**
History of hypertension	–	–	1.286 (1.014–1.632)	**0**.**038**
Past smoker	–	–	1.261 (1.029–1.544)	**0**.**025**
NRP	–	–	0.365 (0.248–0.538)	**<0**.**001**

^a^ ALT values were skewed, ALT values were log transformed before inclusion in the model. Therefore, OR values refer to a unit increase in Log_2_ALT or rather a doubling in ALT. In each case the reported odds ratios are adjusted by our multivariable model. The reported effect of each variable is therefore independent of each other variable in the model.

Data pooled from all 5 imputed data sets (DBD *n* = 3,350, DCD *n* = 2,074 per imputation).

DBD, donation after brain death; DCD, donation after circulatory death; OR, odds ratio; CI, confidence interval; ALT, alanine transaminase; CVA, cerebrovascular accident; NRP, normothermic regional perfusion.

Bold *P* values indicate odds ratios that reached statistical significance.

AST also had a significant effect on liver decline rate in all groups (DBD aOR = 1.457 (95% CI: 1.341–1.582) *p* < 0.001, DCD aOR = 1.218 (95% CI: 1.085–1.369) *p* = 0.003, all donor types aOR = 1.337 (95% CI: 1.236–1.447) *p* < 0.001) (Supplementary Tables S3, S4).

**Figure 1:**
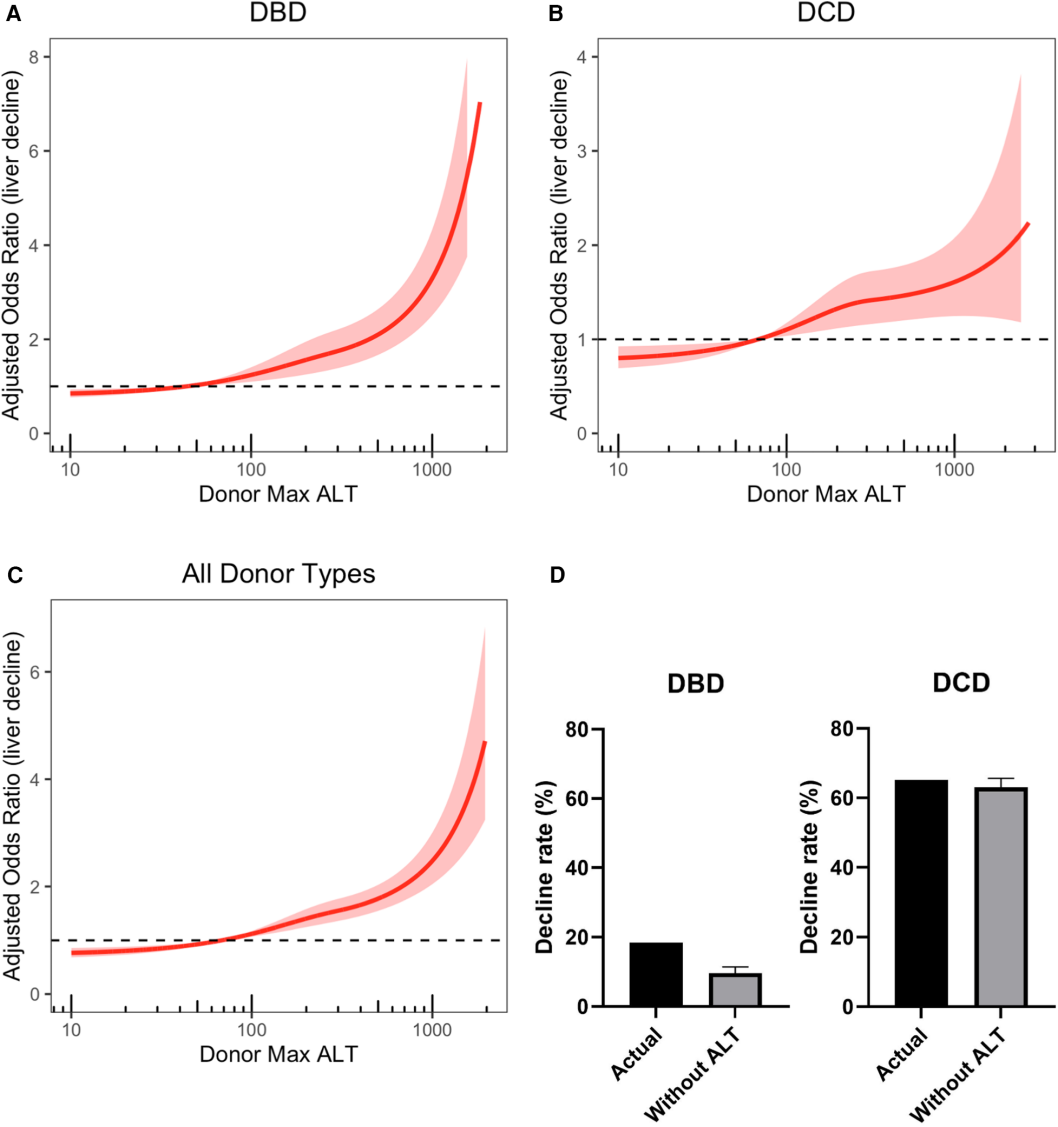
Restricted cubic splines showing the adjusted odds ratio of liver decline with increasing donor max ALT. **(A)** DBD donors only **(B)** DCD donors only **(C)** All donor types. Each restricted cubic spline was generated using 3 knots. Models are adjusted for all cofounders listed in Table 2 **(A,B)** or Supplementary Table S2 **(C)** The shaded area represents the 95% confidence interval and the dashed line at 1 represents no impact on outcome. **(D)** Bar charts showing decline rates for livers with raised ALT in DBD and DCD groups. “Without ALT” represents the decline rate predicted by our model if donor ALT was ignored. DBD, donation after brain stem death; DCD, donation after circulatory death; ALT, alanine transaminase.

Restricted cubic spline modelling was used to represent the relationship between ALT level and organ decline. This suggested that ALT significantly affects decline rate in DBD, DCD and all donor type groups (Figures 1A–C), when adjusting for all the variables shown in Table 2. Sensitivity analyses with the exclusion of donors with ALT values >1,000 U/L were consistent with all of the models discussed above.

### Modelling predicted improvements in utilisation

It has been demonstrated that donor ALT does not influence transplant outcomes (2, 3). Therefore, declining a liver based on donor ALT value represents an unnecessary organ decline, and avoiding this (by not considering ALT when assessing organs) may allow for the safe transplantation of more livers. We developed a methodology to assess impact of avoiding ALT-based organ decline in livers from donors with raised ALT.

18.5% of DBD livers with raised peak ALT (>40) were declined for transplantation. Predicted decline rate in this cohort (adjusted for all factors in Table 2, except ALT), if clinicians had ignored donor ALT in their decision making process, was only 9.6% (95% confidence interval; 7.8%–11.4%); Figure 1D. This corresponds to an absolute decrease in decline rate of 8.9% (7.1%–10.7%), and a relative reduction in DBD decline rate of 48% (38%–58%). This represents an extra 37 (30–45) livers every year that could be safely accepted and transplanted each year; Table 3.

**Table 3:** Modelling impact of avoiding ALT-based organ decline.

	DBD (*n* = 1,683)	DCD (*n* = 1,350)
Actual decline rate (2016–2019)	18.5%	65.2%
Predicted decline rate (95% CI)	9.6% (7.8%–11.4%)	63.1% (60.5%–65.6%)
Absolute decrease in decline (95% CI)	**8.9%** (**7.1%**–**10.7%)**	2.1% (−0.4%–4.7%)
Relative decrease in decline (95% CI)*	48% (38%–58%)	3.2% (0%–7.2%)
No. excess livers declined per year (95% CI)*	**37** (**30–45)**	7 (0–16)

CI, confidence interval; DBD, donation after brain stem death; DCD, donation after circulatory death.

Both models include only livers from donors with peak ALT > 40. Data is averaged across the duration of the study (2016–2019). Predicted decline rate is based on a model including all factors in Table 2, with the exclusion of ALT, and fit to the “normal ALT” (<40 U/L) group. Predicted decline rate calculated for individual liver offer and then averaged. This represents the predicted decline rate, were these donors to have a normal ALT but otherwise identical characteristics. Confidence intervals were calculated with the bootstrap method. Actual and predicted decline rates were then used to calculate decrease in organ decline if no livers were declined based on raised ALT.

Bold values indicate statistical significance, where the 95% CI does not cross 0.

Liver decline rate in the DCD cohort was much higher at 63.1%. The DCD model did not predict a significant decrease in liver decline when donor max ALT was excluded as a predictor [2.13% (−0.42%–4.66%)]; Table 3.

As relatively few donors in our previous study had ALT > 1,000, we cannot be certain that extreme ALT values do not influence outcome (2). Sensitivity analysis revealed no change in significance with the exclusion of donors with ALT values >1,000 U/L (*n* = 142).

## Discussion

It has been demonstrated that donor ALT measurement does not influence transplant outcome, and therefore raised donor ALT should not be used as a reason to decline a liver for transplantation (2, 3). This large cohort study shows that over the same period of time, livers with raised donor peak ALT were more likely to be discarded.

In some cases raised ALT will be the primary reason for organ discard. However, in many cases ALT may represent the “final straw” in the decision-making process, for which an otherwise transplantable liver was declined. Therefore, the impact on utilisation if raised ALT was ignored in the decision-making process cannot be accurately assessed by looking solely at the primary reason for organ discard. We solve this by developing a new methodology to quantify excess organ decline based on raised donor ALT values. In DBD donors with raised peak ALT (>40 U/L), if ALT was removed from this decision-making process organ decline rate would be reduced by 48% (38%–58%), resulting in 37 additional liver transplants each year (averaged from 4 years of offering data).

Some clinicians may use an assessment of ALT trend in utilisation decision-making. However, how each clinician interprets ALT trend will be extremely heterogenous. For example, whether it is rising or falling, absolute change, relative change or rate of change. Throughout this study we focused on the peak ALT, as this is the variable most reported in the literature.

The finding that peak ALT influences clinicians’ decision to accept a liver is in keeping with findings from the UNOS registry (3). Kaltenback et al. performed a multivariable model for graft utilisation, and found that increasing peak ALT was associated with increased organ decline in the US setting, with livers from donors with peak ALT > 500 being 50% less likely to be transplanted. However, this study split their cohort into arbitrary categories (reducing power), and did not perform modelling to assess the number of extra livers which could be transplanted each year (6).

The concern around accepting livers from a donor with raised ALT is exacerbated by current guidelines and definitions. The UK National Health Service defines donors with ALT > 150 U/L as low quality, and even small raises in donor transaminases (ALT > 105 U/L or AST > 90 U/L) are part of the Eurotransplant definition of marginal livers (3, 7–9). Furthermore, the most cited definition of extended criteria liver donors states any elevation of donor ALT or AST as one of the defining criteria (10). Such definitions of extended criteria donors are used in the inclusion criteria of several pivotal clinical trials (11, 12). Updating these definitions to remove small elevations in donor transaminases as a marker of “marginal livers”, based on previous evidence, would be an important step in driving improved utilisation in this group.

One key strength of this work, is that it assesses utilisation over the exact same time frame that we have shown donor liver blood tests do not impact on outcome (2). Another strength is that we have developed a new methodology to quantify how many excess livers would be transplanted, if a specific donor variable is not used as a reason for organ decline. This methodology could be applied to other variables, other settings, and other organ types in the future.

Increasingly, machine perfusion techniques are being employed both experimentally and therapeutically. Although NRP was included as a variable, no data on which livers received ex situ machine perfusion were available, which is the main limitation of our study. Those that received liver perfusion may have been more likely to be accepted for transplantation (13). Those livers with high donor transaminase which may otherwise have been declined, may have been placed on ex situ machine perfusion for viability assessment with subsequent transplantation. Although donor transaminases are not a useful marker of post-transplant outcome, transaminase values during machine perfusion do form the cornerstone of most ex-situ machine perfusion viability criteria (14). DCD livers could have been more likely to undergo ex situ machine perfusion, which may be one explanation for the differences we observed between DCD and DBD; once machine perfusion viability criteria are implemented far less onus will be placed on donor factors (meaning donor ALT will be a less important factor in graft utilisation).

Whilst previous work on transplant outcomes was performed on large data sets, and corroborated between UK and US, it is possible that a small relationship with donor ALT was missed. Although no influence of donor ALT on outcome was demonstrated (2, 3), it is important to note the range of ALT values in these studies. It is relatively uncommon for livers to be transplanted from donors with ALT > 1,000 (*n* = 62 of 3,299 in Tingle et al. and *n* = 1,546 of 59,043 in Kaltenbach et al). Therefore, although there is no evidence of inferior outcomes in this group, as donor ALT values become more extreme, we are less certain that they will not influence outcome. For this reason, we performed a sensitivity analyses, where donors with peak ALT > 1,000 were excluded, and these were in keeping with our main analysis.

In conclusion, we have shown that raised donor ALT values have been used as a reason to decline livers. Based on previous research, we know that raised donor ALT values are not a predictor of outcome, and that such livers can be safely transplanted when other factors are favourable. In this paper we demonstrate that avoiding donor ALT-based organ decline is an immediate and effective way to expand the donor pool.

## Data Availability

The original contributions presented in the study are included in the article/Supplementary Material, further inquiries can be directed to the corresponding author.
