# Supplementary material for: Impact of donor transaminases on liver transplant utilisation and unnecessary organ discard: national registry cohort study
Source: Front Transplant. 2024 Sep 4;3:1458996. doi: 10.3389/frtra.2024.1458996 (PMC11421386; doi:10.3389/frtra.2024.1458996)
Supplement: Supplementary file 1 [file Table1.docx]

# Supplementary tables

*Supplementary Table 1- Summary of Missing Data*

| **Variable** | **N** | **Missing** | **% Missing** |
| --- | --- | --- | --- |
| **Donor Type** | 5424 | 0 | 0 |
| **NRP** | 5424 | 0 | 0 |
| **Cause of Death** | 5410 | 14 | 0.3 |
| **Donor Age** | 5424 | 0 | 0 |
| **Donor Sex** | 5424 | 0 | 0 |
| **Donor BMI** | 5399 | 25 | 0.5 |
| **Year of Donation** | 5424 | 0 | 0 |
| **Donor Blood Group** | 5424 | 0 | 0 |
| **Liver Decline** | 5424 | 0 | 0 |
| **History of Cardiac Disease** | 5296 | 128 | 2.4 |
| **History of Diabetes** | 5386 | 38 | 0.7 |
| **Family History of Diabetes** | 5167 | 257 | 4.7 |
| **History of Hypertension** | 5345 | 79 | 1.5 |
| **History of Liver Disease** | 5324 | 100 | 1.8 |
| **Smoking History** | 5397 | 27 | 0.5 |
| **Donor Max ALT** | 5251 | 173 | 3.2 |
| **Donor Max AST** | 2011 | 3413 | 62.9 |

*NRP, normothermic regional perfusion; ALT, alanine transaminase; AST, aspartate aminotransferase.*

*Supplementary Table 2 - Multiple logistic regression model showing the effect of donor max ALT on utilisation across all donor types.*

| **Variable** | **OR(95% CI)** | **P value** |
| --- | --- | --- |
| **Donor max ALT (log_2_ALT)** | 1.279 (1.218-1.342) | **<0.001** |
| **Donor type** |  |  |
| **DBD** | 1 | - |
| **DCD** | 11.474 (9.875-13.333) | **<0.001** |
| **NRP** | 0.356 (0.242-0.524) | **<0.001** |
| **Cause of death** |  |  |
| **CVA** | 1 | - |
| **Trauma** | 0.805 (0.529-1.225) | 0.312 |
| **Hypoxia** | 0.771 (0.643-0.926) | **0.005** |
| **Other** | 1.227 (0.971-1.550) | 0.086 |
| **Donor age (years)** | 1.022 (1.017-1.028) | **<.001** |
| **Donor sex** | 0.834 (0.723-0.963) | **0.013** |
| **Donor BMI (kg/m^2^)** | 1.085 (1.070-1.099) | **0.000** |
| **Year of donation (years)** | 1.155 (1.085-1.229) | **<0.001** |
| **Donor blood group** |  |  |
| **O** | 1 | **-** |
| **A** | 1.160 (1.001-1.345) | **0.049** |
| **B** | 1.227 (0.953-1.581) | 0.113 |
| **AB** | 3.541 (2.420-5.180) | **<0.001** |
| **History of cardiac disease** | 1.282 (1.045-1.572) | **0.017** |
| **History of hypertension** | 1.159 (0.982-1.370) | 0.082 |
| **History of diabetes** | 1.447 (1.125-1.862) | **0.004** |
| **Family history of diabetes** | 1.158 (0.994-1.350) | 0.060 |
| **History of liver disease** | 5.233 (3.389-8.080) | **<0.001** |
| **Past smoker** | 1.235 (1.067-1.429) | **0.005** |

*aALT values were skewed, ALT values were log transformed before inclusion in the model. Therefore, OR values refer to a unit increase in Log_2_ALT or rather a doubling in ALT.*

*Data pooled from all 5 imputed data sets (n=5424 per imputation).*

*DCD, donation after circulatory death; OR, odds ratio; CI, confidence interval; ALT, alanine transaminase; CVA, cerebrovascular accident;*

*Supplementary Table 3 - Multiple logistic regression showing the effect of donor AST on liver decline in DBD and DCD donors*

| **Variable** | **DBD** | | **DCD** | |
| --- | --- | --- | --- | --- |
|  | **OR(95% CI)** | **P value** | **OR(95% CI)** | **P value** |
| **Donor max AST (Log_2_AST)** ***^a^*** | 1.457 (1.341-1.582) | **<0.001** | 1.218 (1.085-1.369) | **0.003** |
| **Cause of death** |  |  |  |  |
| **CVA** | 1 | **-** | **1** | **-** |
| **Trauma** | 0.789 (0.414-1.504) | 0.472 | 0.858 (0.500-1.472) | 0.577 |
| **Hypoxia** | 0.460 (0.341-0.620) | **<0.001** | 1.114 (0.870-1.427 | 0.390 |
| **Other** | 0.830 (0.569-1.212) | 0.335 | 1.795 (1.299-2.482) | **<0.001** |
| **Age (years)** | 1.018 (1.011-1.026) | **<0.001** | 1.023 (1.016-1.031) | **<0.001** |
| **Sex** |  |  |  |  |
| **Male** | 1 | **-** | **-** | **-** |
| **Female** | 0.773 (0.628-0.952) | **0.015** | **-** | **-** |
| **Donor BMI (kg/m^2^)** | 1.086 (1.066-1.107) | **<0.001** | 1.104 (1.082-1.126) | **<0.001** |
| **Year of donation (years)** | 1.171 (1.066-1.285) | **<0.001** | 1.166 (1.070-1.271) | **<0.001** |
| **Donor blood group** |  |  |  |  |
| **O** | 1 | - | 1 | - |
| **A** | 1.122 (0.898-1.401) | 0.312 | 1.233 (1.004-1.514) | **0.046** |
| **B** | 1.325 (0.934-1.879) | 0.115 | 1.195 (0.825-1.731) | 0.345 |
| **AB** | 4.240 (2.658-6.762) | **<0.001** | 2.563 (1.377-4.772) | **0.003** |
| **History of diabetes** | 1.716 (1.233-2.389) | **0.001** | - | - |
| **Family history of diabetes** | 1.397 (1.118-1.744) | **0.003** | - | - |
| **History of liver disease** | 6.219 (3.712-10.418) | **<0.001** | 2.526 (1.245-5.123) | **0.010** |
| **History of cardiac disease** | - | **-** | 1.403 (1.044-1.886) | **0.025** |
| **History of hypertension** | - | - | 1.292 (1.015-1.644) | **0.037** |
| **Past smoker** | - | - | 1.260 (1.023-1.551) | **0.030** |
| **NRP** | - | - | 0.374 (0.253-0.552) | **<0.001** |

*aAST values were skewed, AST values were log transformed before inclusion in the model. Therefore, OR values refer to a unit increase in Log_2_ALT or rather a doubling in ALT.*

*Data pooled from all 5 imputed data sets (DBD n=3350, DCD n=2074 per imputation).*

*DBD, donation after brain death; DCD, donation after circulatory death; OR, odds ratio; CI, confidence interval; ALT, alanine transaminase; CVA, cerebrovascular accident; NRP, normothermic regional perfusion.*

*Supplementary Table 4 - Multiple logistic regression for liver decline in all donor types, showing the effect of donor max AST*

| **Variable** | OR (95% CI) | **P value** |
| --- | --- | --- |
| **Donor max AST (Log_2_AST)** | 1.337 (1.236-1.447) | **<0.001** |
| **Donor type** |  |  |
| **DBD** | 1 | **-** |
| **DCD** | 11.382 (9.763-13.269) | **<0.001** |
| **NRP** | 0.372 (0.252-0.550) | **<0.001** |
| **Cause of death** |  |  |
| **CVA** | 1 | **-** |
| **Trauma** | 0.773 (0.498-1.199) | 0.249 |
| **Hypoxia** | 0.774 (0.642-0.933) | **0.007** |
| **Other** | 1.265 (0.999-1.603) | 0.051 |
| **Donor age (years)** | 1.022 (1.016-1.027) | **<0.001** |
| **Donor sex** |  |  |
| **Male** | 1 | **-** |
| **Female** | 0.823 (0.712-0.951) | **0.008** |
| **Donor BMI (kg/m^2^)** | 1.090 (1.076-1.105) | **<0.001** |
| **Year of donation (years)** | 1.166 (1.095-1.241) | **<0.001** |
| **Donor blood group** |  |  |
| **O** | 1 | **-** |
| **A** | 1.175 (1.011-1.366) | **0.035** |
| **B** | 1.253 (0.970-1.619) | 0.084 |
| **AB** | 3.531(2.395-5.206) | **<0.001** |
| **History of cardiac disease** | 1.239 (1.008-1.524) | **0.042** |
| **History of hypertension** | 1.177 (0.992-1.396) | 0.062 |
| **History of diabetes** | 1.430 (1.110-1.843) | **0.006** |
| **Family history of diabetes** | 1.189 (1.018-1.388) | **0.029** |
| **History of liver disease** | 4.904 (3.147-7.642) | **<0.001** |
| **Smoking history** | 1.221 (1.048-1.422) | **0.010** |

*aAST values were skewed, AST values were log transformed before inclusion in the model. Therefore, OR values refer to a unit increase in Log_2_AST or rather a doubling in AST.*

*Data pooled from all 5 imputed data sets (n=5424 per imputation).*

*DBD, donation after brain stem death; DCD, donation after circulatory death; OR, odds ratio; CI, confidence interval; AST, aspartate aminotransferase; CVA, cerebrovascular accident;*

*Supplementary Table 5 - Liver decline rate*

|  | **Decline Rate** | |
| --- | --- | --- |
|  | **DBD** | **DCD** |
| **Normal Donor ALT** | 11.0% | 59.1% |
| **Raised Donor ALT** | 18.5% | 65.2% |
| **All Donors** | 14.9% | 63.2% |

*Shows the decline rate of livers in donors which donated at least one other organ. DBD All donors n=3234, normal donor ALT n= 1551, raised donor ALT n= 1683. DCD all donors n=2017, normal donor ALT n= 667, raised donor ALT n= 1350.*
